# Patient-Reported Knee Function and Return-to-Sport Rates After Nonsurgical and Surgical Treatment of an Acute Anterior Cruciate Ligament Injury: Results From the NACOX Prospective Cohort Study

**DOI:** 10.1177/03635465261451698

**Published:** 2026-06-08

**Authors:** Anna Croné, Håkan Gauffin, Henrik Hedevik, Anne Fältström, Joanna Kvist

**Affiliations:** † Department of Orthopaedics and Department of Biomedical and Clinical Sciences, Linköping University, Linköping, Sweden; ‡ Center for Medical Image Science and Visualization (CMIV), Department of Health, Medicine and Caring Sciences, Linköping University, Linköping, Sweden; § Unit of Physiotherapy, Department of Health, Medicine and Caring Sciences, Linköping University, Linköping, Sweden; ‖ Region Jönköping County Rehabilitation Centre, Ryhov County Hospital, Jönköping, Sweden; Investigation performed at Linköping University, Linköping, Sweden

**Keywords:** ACL injury, ACL reconstruction, return to sport, IKDC, nonoperative treatment

## Abstract

**Background:**

The best treatment for acute anterior cruciate ligament (ACL) injuries (surgical or nonsurgical) remains uncertain, particularly regarding return to sport (RTS) and knee function.

**Purpose:**

To compare patient-reported knee function (International Knee Documentation Committee Subjective Knee Form [IKDC-SKF]) and RTS between non-ACL reconstruction (ACLR) and ACLR treatment strategies 24 months after ACL injury or reconstruction and identify predictors of these outcomes.

**Study Design:**

Cohort study; Level of evidence, 2.

**Methods:**

From the NACOX multicenter cohort, 272 patients (mean age, 25.5 years; 95% CI, 24.6-26.3 years; 52% females) with acute ACL injuries were followed for 24 months after ACL injury or ACLR. Treatment (non-ACLR vs ACLR) was determined via shared decision-making in routine practice. IKDC-SKF scores were collected at 3, 6, 12, and 24 months and analyzed using a linear mixed-effects model adjusted for age, sex, preinjury activity level, and new serious knee injury. RTS was defined as return to preinjury Tegner level or higher. Risk factors for not returning to sport were evaluated using log-binomial regression.

**Results:**

The ACLR group had higher preinjury Tegner levels (8 vs 6.5; *P* < .001) and was younger (24.2 vs 27.6 years; *P* < .001). No differences in IKDC-SKF scores were observed between non-ACLR and ACLR groups. IKDC-SKF scores improved over time for both groups (*P* < .001) but were negatively affected by new serious knee injury (*P* < .001), older age (*P* = .003), and female sex (*P* = .004). Overall, 75% of patients achieved RTS within 24 months (mean, 6.3 months; 95% CI, 5.6-7.0 months), with no difference between groups (72% non-ACLR vs 77% ACLR; *P* = .286). Patients without ACLR returned to sport earlier (3.5 months [95% CI, 2.8-4.2 months] vs 8.1 months [95% CI, 7.2-9.0 months]; *P* < .001). In patients aged 26 to 40 years, ACLR was associated with a 3.15-fold higher risk of not returning to sport at 24 months compared to the non-ACLR group (*P* = .015).

**Conclusion:**

In this cohort of patients with ACL injury, both nonsurgical management and ACLR, determined through shared decision-making, resulted in comparable patient-reported knee function and RTS rates at 24 months, although the ACLR group was younger and had higher activity level. Non-ACLR treatment may therefore be a viable option for selected patients, particularly those who are older or have lower activity demands, to achieve satisfactory RTS outcomes. Older age (26-40 years) was associated with a higher risk of not returning to sport after ACLR, highlighting the importance of individualized treatment decisions.

Anterior cruciate ligament (ACL) injuries are severe knee injuries, mainly seen in young physically active athletes in pivoting sports. The injury could lead to unsatisfactory knee function and may adversely affect long-term participation in sport.^7,31^

Treatment for ACL injury can be rehabilitation alone or in combination with ACL reconstruction (ACLR) and aims to restore knee function, prevent new injuries, and, in many cases, make it possible for the athlete to return to sport (RTS).^7,13^ However, there is conflicting evidence as to whether RTS rates and knee patient-reported outcome measures (PROMs) differ between non-ACLR and ACLR treatment. Some studies showed no difference in PROMs or RTS rates between these treatment strategies,^9,10,13,21,22^ while others found higher RTS rates after ACLR.^14,18^ Some predictors for better PROMs are early/preoperative quadriceps strength and hop symmetry,^13^ and new knee injuries are associated with worse PROMs, independently of treatment strategy.^13^ Studies on patients with ACLR have reported several predictors of better PROMs, including younger age, male sex,^20^ ACLR with hamstring tendon graft (compared with bone–patellar tendon–bone), and complete adherence to a rehabilitation program.^19,23,25^ In contrast, factors associated with lower PROMs include meniscal resection and concomitant meniscal or cartilage injuries.^6,7,23^

RTS after ACL injury remains a central outcome for both athletes and clinicians. For patients who wish to return to linear activities (eg, running, weight lifting, cycling), nonsurgical treatment could be an option.^7^ However, ACLR is recommended in active patients who want to return to pivoting sports, patients with concomitant meniscal injuries requiring surgery or multiple ligament injuries, and patients with persistent instability in daily living activities.^7^ Reported RTS rates are between 46% and 68% after non-ACLR treatment.^14,28^ Reported RTS rates after ACLR vary widely, ranging from as low as 16% to nearly 90% depending on study design, follow-up time, and population characteristics.^2,3,16,18,24,29,31,32^ Higher rates of RTS after ACLR occurred, in general, in elite athletes or high-activity patients.^16,18,24,29,31^ Other predictors for RTS after ACLR have been suggested to be younger age,^3^ male sex, and positive psychological response, such as confidence in the knee and reduced fear of reinjury.^1,7^ Recently, a systematic review and meta-analysis showed no differences in RTS rate in patients treated without or with ACLR,^8^ although the included studies had some limitations due to lack of data regarding the time of RTS and activity level. In addition, the definition of RTS differs between studies, as do follow-up times.

Despite numerous studies, it remains unclear whether PROMs and RTS differ between patients treated nonsurgically or treated with ACLR during the first 24 months after injury or ACLR, emphasizing the need for studies with a mixed, real-world cohort with standardized outcomes to guide clinical decision-making. By providing direct comparisons across treatment strategies using validated PROMs and standardized follow-up, the present study aimed to generate clinically meaningful suggestions to guide treatment decision-making for individuals with ACL injury.

The aim of this study was to (1) compare PROMs (International Knee Documentation Committee Subjective Knee Form [IKDC-SKF]) and RTS during 24 months after ACL injury or ACLR, and (2) investigate whether factors (age, sex, preinjury Tegner activity level, new serious knee injury) influenced PROMs and RTS in these 2 groups.

## Methods

### Study Design and Patients

This study is part of the Natural Corollaries and Recovery after Acute ACL Injury (NACOX) study,^17^ a multicenter, prospective cohort study including patients who sustained an acute ACL injury between 2016 and 2018, from 7 sites in Sweden (mix of public and private health care clinics). The inclusion criteria were patients between 15 and 40 years of age with an acute ACL injury (within 6 weeks). The exclusion criteria were a previous ACL injury in the same knee, a serious concomitant injury (fracture that requires separate treatment), an inability to understand written and spoken Swedish, a cognitive impairment, or any other illness or injury that impairs function (eg, fibromyalgia, rheumatic diseases, or other diagnoses associated with chronic pain).

Participation in the study did not alter the usual course of treatment for patients with an ACL injury at recruiting centers. Some of the patients underwent ACLR after the index ACL injury at some point, and some continued with rehabilitation without ACLR. This was determined through a shared decision-making process, as described previously for a subgroup of the cohort.^11,12^ All patients received study information and gave their written consent to participate in this study. The Swedish Ethical Review Authority has approved the NACOX study (Dnr. 2016/44-31 and 2017/221-32).

### Data Collection

Patients included in the NACOX study were confirmed to have ACL rupture through clinical evaluation by an orthopaedic surgeon and, in most cases, by an MRI study obtained at baseline. An MRI study was not obtained in 21 of the 275 patients (7.6%) at the time of inclusion. Of these, 18 patients subsequently underwent ACLR (mean, 5.1 months from ACL injury to ACLR), confirming the diagnosis intraoperatively. The remaining 3 patients were treated nonoperatively and were diagnosed based on clinical examination. These patients were excluded from the present analyses due to lack of MRI confirmation of the ACL rupture and thus uncertainty regarding structural diagnosis.

Digital questionnaires were sent out regularly (initially weekly, later monthly) regarding knee function, symptoms, physical activity participation, reinjuries, and surgeries of the knee. The IKDC-SKF, reporting knee symptoms and function, was answered at baseline and at 3, 6, 12, and 24 months after injury or after ACLR. Activity level and sports participation were classified according to the Tegner score^30^ and IKDC sports level.^15^

If data were missing by 2 years of follow-up due to response failure, patients were contacted via telephone, letter, or email. Medical charts were reviewed to investigate whether the patient had visited a health care clinic due to a new knee injury, and patients were also checked for having undergone ACLR in the Swedish Knee Ligament Registry.^5^

### Group Definitions: Non-ACLR and ACLR

Our cohort was divided into 2 groups: non-ACLR and ACLR. The non-ACLR group consisted of those patients who had not undergone ACLR within 2 years after index ACL injury. Their baseline was the time of the index ACL injury. The ACLR group constituted those patients who had undergone an ACLR within 2 years of the index ACL injury. Their baseline was the time of the ACLR.

### RTS Definition

The definition for RTS was when the patient had returned to their preinjury Tegner sports level or higher, based on patients’ self-reported main sports before the ACL injury.

### Classification of Knee Reinjuries

We defined knee reinjury as an increase in knee symptoms or new symptoms from the knee as a result of a traumatic injury during an activity-related exposure.^26,27^ All reported reinjuries, including graft ruptures, were classified as reinjury not leading to surgery or reinjury leading to surgery.^26^

### Statistical Analysis

All statistical analyses were performed using IBM SPSS Statistics for Windows (Version 29) and R (Version 4.4.1; R Foundation for Statistical Computing), using the packages survival (Version 3.7-0), ggsurvfit (Version 1.1.0), ggplot2 (Version 3.5.1), and ggsci (Version 3.1.0). Descriptive statistics are reported as means with 95% confidence intervals (CIs) for normally distributed continuous variables, medians with interquartile ranges (IQRs) for nonnormally distributed variables, and numbers with percentages for categorical variables. Baseline patient characteristics were compared between the non-ACLR and ACLR groups using independent-samples *t* tests for normally distributed continuous variables, Mann-Whitney *U* tests for ordinal variables, and chi-square tests for nominal variables. The Fisher exact test was used when >20% of expected cell counts were <5.

IKDC-SKF scores at 3, 6, 12, and 24 months were analyzed using a linear mixed-effects model (LMM) with fixed effects for time (categorical), treatment group (non-ACLR vs ACLR), and their interaction (time × group), adjusted for age, sex, preinjury Tegner activity level, and new serious knee injury. An unstructured covariance structure accounted for within-patient correlation. Estimated marginal means and adjusted mean differences with 95% confidence intervals were obtained. The method of Satterthwaite was used for degrees of freedom. Prespecified contrasts were used to test whether between-group differences changed over time.

We estimated the risk ratio for not returning to sport at 24 months comparing non-ACLR and ACLR using log-binomial regression. Model-based Wald 95% confidence intervals and *P* values are reported. Analyses were performed in the total cohort and stratified for sex, age interval, and preinjury Tegner activity level.

Time to RTS was evaluated using Kaplan-Meier survival analysis, and group differences were assessed using the log-rank test. Additional stratified Kaplan-Meier analyses were performed for age, sex, and preinjury Tegner activity level.

To assess the potential influence of missing data, a nonresponse analysis was conducted comparing baseline characteristics between participants included in the LMM and those who were excluded.

A *P* value <.05 was considered statistically significant.

## Results

### Patient Characteristics

This study included 272 patients (52% female) with a mean age of 25.5 years (95% CI, 24.6-26.3 years) (Table 1). At 24 months of follow-up, 170 patients had undergone an ACLR^11,12^ at a mean of 5.6 months (95% CI, 5.0-6.2 months) from the index injury (Appendix Figure A1, available in the online version of this article). The grafts used were hamstring (n = 150; 88%), patellar tendon (n = 18; 11%), and quadriceps tendon (n = 2; 1%). A majority (n = 190; 70%) were active in pivoting or contact sports (IKDC activity level 1 or 2) before the injury. The ACLR group was younger and had a higher preinjury Tegner activity level compared to the non-ACLR group (Table 1). Almost all patients (96%) had a goal of returning to sport.

**Table 1:** Patient Characteristics at Baseline and Follow-up Data Within 24 Months From ACL Injury or ACLR*^*a*^*

	Total (N = 272)	Non-ACLR (n = 102)	ACLR (n = 170)	*P* Value
Baseline data				
Age, y	25.5 (24.6-26.3)	27.6 (26.1-29.0)	24.2 (23.2-25.2)	<.001
Sex				.117
Male	130 (48)	55 (54)	75 (44)	
Female	142 (52)	47 (46)	95 (56)	
Body mass index, kg/m^2^	23.9 (23.5-24.3)^1^	24.4 (23.7-25.2)	23.6 (23.2-24.0)^1^	.227
Concomitant injuries*^*b*^*	189 (74)^18^	76 (75)	113 (74)^18^	.976
Meniscal injury	164 (65)^19^	62 (61)^1^	102 (67)^18^	.351
Ligament injury	44 (17)^18^	25 (25)	19 (13)^18^	.013
Cartilage injury	34 (13)^18^	18 (18)	16 (11)^18^	.102
Preinjury Tegner activity level	7 (4, 9)	6.5 (4, 9)	8 (4, 9)	<.001
Preinjury IKDC activity level*^*c*^*				<.001
Level 1	157 (58)	44 (43)	113 (66)	
Soccer	98 (62)	27 (61)	71 (63)	
Floorball	32 (20)	12 (27)	20 (18)	
Handball	17 (11)	2 (5)	15 (13)	
Other contact/pivoting sports*^*d*^*	10 (6)	3 (7)	7 (6)	
Level 2	33 (12)	14 (14)	19 (11)	
Level 3	82 (30)	44 (43)	38 (22)	
Follow-up data within 24 mo				
New serious injury within 2 y	32 (12)	6 (6)	26 (15)	.020
Serious injury	5 (2)	2 (2)	3 (2)	
Serious injury, leading to surgery	9 (3)	4 (4)	5 (3)	
Graft rupture*^*e*^*	15 (6)	NA	15 (9)	
Contralateral injury	3 (1)	00 (0)	3 (2)	
RTS Tegner activity level	190 (75)^19^	73 (72)	117 (77)^19^	.286
Months to RTS	6.3 (5.6-7.0)	3.5 (2.8-4.2)	8.1 (7.2-9.0)	<.001

a Data are presented as n (%), mean (95% CI), or median (IQR). Superscript numbers indicate missing values. ACL, anterior cruciate ligament; ACLR, anterior cruciate ligament reconstruction; IKDC, International Knee Documentation Committee; NA, not applicable; RTS, return to sport.

b Concomitant injuries (medial and/or lateral meniscus, total ligament [medial collateral ligament and/or lateral collateral ligament] rupture, partial or full cartilage lesion) in the ipsilateral knee joint.

c IKDC level: 1, contact and pivoting sports; 2, noncontact but pivoting sports; 3, neither contact nor pivoting sports.

d Basketball, ice hockey, American football, rugby.

e Six of the graft ruptures led to revision surgery (5 quadriceps tendon and 1 patellar tendon), and 3 were partial graft ruptures.

### Patient-Reported Knee Function

The IKDC-SKF score improved over time in both groups (*P* < .001), with no statistically significant difference between the 2 groups at any time point after ACL injury or ACLR (Table 2, A and *B*).

**Table 2a:** Linear Mixed-Effects Adjusted*^*a*^* Model on IKDC-SKF Score by Time and Group*^*a*^*

	Numerator df	Denominator df	*F*	*P* Value	Estimate (95% CI)
Time	3	158.352	174.516	<.001	
Group	1	208.588	0.072	.789	
Time × group	3	156.469	3.036	.031	
Female sex	1	212.221	8.383	.004	−5.6 (−9.4 to −1.8)
Age at injury	1	211.368	9.182	.003	−0.4 (−0.7 to −0.1)
Preinjury Tegner activity level	1	210.519	0.094	.759	0.1 (−0.7 to 0.9)
New serious knee injury	1	407.456	19.584	<.001	−12.4 (−17.9 to −6.9)

a Covariates in model: age (mean, 25.9 years), sex (56.0% females), preinjury Tegner activity level (mean, 6.4), new serious injury (4.0%). df, degrees of freedom; IKDC-SKF, International Knee Committee Documentation Subjective Knee Evaluation Form.

**Table 2b:** Estimated Marginal Means From the Linear Mixed-Effects Adjusted Model on IKDC-SKF Score by Time and Group*^*a*^*

	Total (N = 230)	Non-ACLR (n = 93)	ACLR (n = 137)	Difference: Non-ACLR − ACLR
IKDC-SKF score, %				
3 mo	55.7 (53.7 to 57.8)	56.1 (52.9 to 59.3)	55.3 (52.6 to 58.1)	0.80 (−3.52 to 5.11)
6 mo	68.4 (66.3 to 70.6)	69.8 (66.4 to 73.2)	67.0 (64.2 to 69.9)	2.76 (−1.79 to 7.30)
12 mo	75.9 (73.6 to 78.1)	75.6 (72.0 to 79.1)	76.1 (73.1 to 79.1)	−0.54 (−5.30 to 4.23)
24 mo	77.9 (75.1 to 80.7)	75.3 (71.0 to 79.7)	80.5 (76.9 to 84.2)	−5.21 (−10.93 to 0.52)

a Data are presented as mean (95% CI). Covariates in model: age (mean, 25.9 years), sex (56.0% females), preinjury Tegner activity level (mean, 6.4), new serious injury (4.0%). ACLR, anterior cruciate ligament reconstruction; IKDC-SKF, International Knee Documentation Committee Subjective Knee Form.

In the overall cohort, results from the LMM (Table 2, A and B) indicate that higher age (*P* = .003), female sex (*P* = .004), and sustaining a new serious knee injury (*P* < .001) were significantly associated with lower IKDC-SKF scores, averaged across all time points and both groups.

### Return to Sport

**Figure 1.**
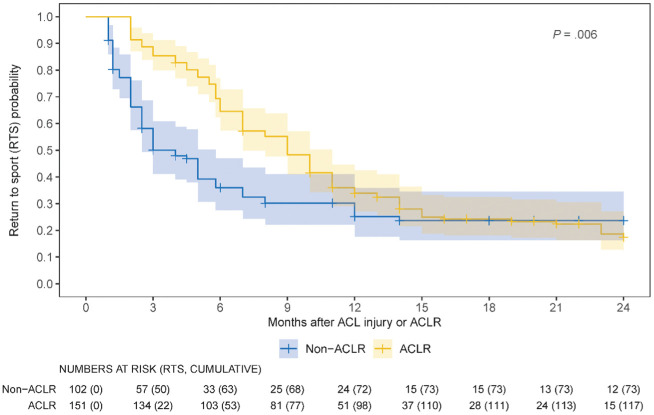
Kaplan-Meier curves on return to sport (same Tegner level or higher) within 24 months by group (non– anterior cruciate ligament reconstruction [ACLR] and ACLR). Months after anterior cruciate ligament (ACL) injury for the non-ACLR and after ACLR for the ACLR group.

In the entire cohort, 75% had returned to sport within 24 months, in a mean of 6.3 months from index ACL injury or ACLR, for those who did return. No difference in RTS rates was found between the non-ACLR and ACLR groups at 24 months (72% vs 77%; *P* = .286) (Table 1). The Kaplan-Meier analysis showed a significant difference in timing for RTS between the 2 groups (*P* = .006) (Figure 1). The non-ACLR group had earlier RTS compared to the ACLR group (3.5 vs 8.1; *P* < .001) (Table 1).

**Figure 2.**
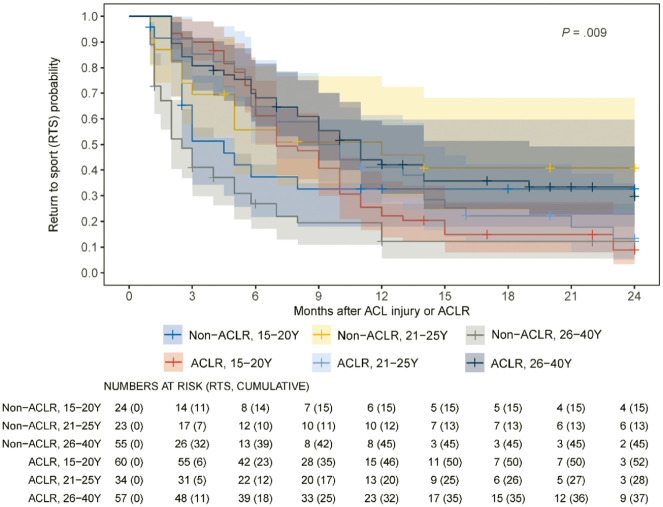
Kaplan-Meier curves on return to sport (same Tegner level or higher) within 24 months by group (non– anterior cruciate ligament reconstruction [ACLR] and ACLR) and stratified by age interval. ACL, anterior cruciate ligament.

Of the assessed factors—sex, age, and pre-injury Tegner activity level—only older age (age interval, 26-40 years) resulted in a different risk for not returning to sport between the groups. In the ACLR group, the older patients had >3.1 times higher risk (*P* = .015) of not returning to sport at 24 months of follow-up compared to the non-ACLR group (Table 3, Figure 2). The RTS rates within 24 months in the ACLR and non-ACLR groups, stratified by sex and preinjury Tegner activity level, can be found in the appendix (Figures A2 and A3, available online).

**Table 3:** Risk Analysis of Not Returning to Sport (Same Tegner Level or Higher) Within 24 Months by Group*^*a*^*

	No.	Risk of Not Returning to Sport, % (95% CI)	Risk Ratio (95% CI)	*P* Value
Group				
Non-ACLR	92	20.7 (13.8-30.8)	1	
ACLR	146	19.9 (14.3-27.5)	0.96 (0.57-1.61)	.882
Sex				
Male				
Non-ACLR	49	22.4 (13.3-37.8)	1	
ACLR	58	22.4 (13.9-36.2)	1.00 (0.49-2.03)	.997
Female				
Non-ACLR	43	18.6 (10.0-34.8)	1	
ACLR	88	18.2 (11.7-28.3)	0.98 (0.45-2.10)	.953
Age interval				
15-20 years				
Non-ACLR	21	28.6 (14.5-56.2)	1	
ACLR	59	11.9 (5.9-23.8)	0.42 (0.16-1.10)	.076
21-25 years				
Non-ACLR	21	38.1 (22.1-65.7)	1	
ACLR	33	15.2 (6.8-34.0)	0.40 (0.15-1.05)	.064
26-40 years				
Non-ACLR	50	10.0 (4.4-23.0)	1	
ACLR	54	31.5 (21.2-46.7)	3.15 (1.25-7.90)	.015
Tegner activity level				
Tegner scores 2-6				
Non-ACLR	49	4.1 (1.1-15.9)	1	
ACLR	40	5.0 (1.3-19.3)	1.23 (0.18-8.31)	.835
Tegner scores 7-9				
Non-ACLR	43	39.5 (27.3-57.2)	1	
ACLR	106	25.5 (18.4-35.3)	0.64 (0.39-1.05)	.080

a ACLR, anterior cruciate ligament reconstruction.

### Nonresponse Analysis

A nonresponse analysis was performed to evaluate potential differences between patients included in the LMM analysis and those excluded due to missing questionnaire data. The total study cohort consisted of 272 patients, of whom 230 had complete data for at least 1 time point and were included in the LMM analysis of IKDC-SKF. There was a significant difference between the 2 groups in response rate; 9 (8.8%) of the non-ACLR vs 33 (19.4%) of the ACLR were nonresponders, and more men were nonresponders in the ACLR group.

## Discussion

At 24 months of follow-up in this prospective cohort study of patients with ACL injury, we found no differences in either patient-reported function (IKDC-SKF) or RTS rate between the non-ACLR and ACLR groups. The non-ACLR group had a lower preinjury activity level, was older, and had earlier RTS compared to the ACLR group. Older patients (26-40 years) with ACLR had >3 times higher risk for not returning to sport at 24 months compared to those without ACLR in the same age interval. Different factors affected the IKDC-SKF score negatively in both treatment groups: female sex, higher age, and new serious knee injury. To guide evidence-based decision-making for patients with ACL injuries, it is crucial to understand how nonsurgical and surgical management influence patient-reported knee functions as measured by the IKDC-SKF and RTS.

We found no difference in IKDC-SKF scores between the non-ACLR group and ACLR group within 24 months. Similar findings have previously been described in a randomized controlled study and a prospective cohort study at both 2 and 5 years of follow-up, suggesting that subjective outcomes can be achieved with either treatment.^9,10,13,21,22^ We found a significant interaction between group and time, which could suggest that the recovery process differs between the groups. Patients undergoing ACLR may experience a more gradual but steady improvement in function during postoperative rehabilitation, whereas the non-ACLR group may reach their functional plateau earlier. This difference in recovery trajectories is clinically relevant and may influence how patients are counseled regarding expected outcomes. However, as previously described, short-term follow-up did not find a difference between the 2 groups in IKDC-SKF score.^9,13^

In the present study, several factors negatively influenced IKDC-SKF outcome in both groups, including sustaining a new serious knee injury, higher age, and female sex. Previous studies of patients with ACLR have reported similar factors, that is, new knee injuries and age affecting the IKDC-SKF score.^13,19,23^ The association between higher age and lower IKC-SKF score may reflect differences in activity level, recovery potential, or underlying joint health that influence the patient's perception of knee function. In our study, the non-ACLR group were older and had lower preinjury Tegner scores, which may partly explain the observed age-related differences in IKDC-SKF score. Female sex was also associated with lower IKDC-SKF score, as previously described.^20^ These findings highlight the importance of considering patient characteristics when counseling and managing expectations regarding knee function.

Sustaining a new serious knee injury was associated with worse IKDC-SKF score. In our cohort, the ACLR group had significantly more new knee injuries compared to the non-ACLR group. We have previously reported similar findings in this cohort when analyzing new injuries from the time of ACL injury, whereas the current analysis used the time from injury or time from ACLR as reference.^26^ In our previous study, preinjury activity level was not identified as a risk factor for new knee injuries. Nevertheless, patients in the ACLR group had higher preinjury Tegner activity levels and a greater proportion participated in pivoting sports, which may reflect greater exposure to high-risk activities. The higher rate of new knee injuries in the ACLR group may therefore contribute to the lower IKDC-SKF scores observed in patients sustaining a subsequent injury. This underscores the need for careful long-term follow-up and structured injury prevention strategies after ACLR and highlights that new knee injuries may partly explain reduced PROMs in clinical practice. Thus, both patient-related and injury-related factors contribute to subjective knee function over the 2-year follow-up period, regardless of whether ACLR is performed. To our knowledge, no prior studies have reported factors influencing IKDC-SKF score in a non-ACLR population. These findings may reveal previously unreported factors of potential clinical relevance.

Both treatment groups in our study demonstrated similar RTS rates at 24 months of follow-up, supporting previous studies comparing non-ACLR and ACLR treatment.^8,13^ In a meta-analysis, Filbay et al^8^ reported no difference in RTS rates between patients treated without and with ACLR, although the quality of evidence of the included studies was rated as low to very low. Similarly, the Delaware-Oslo ACL cohort^13,21,22^ and the KANON (Knee Anterior Cruciate Ligament, Nonsurgical versus Surgical Treatment) trial^8,9^ showed that nonoperatively managed patients can achieve functional and sports activity levels comparable to those who undergo ACLR. In contrast, earlier meta-analyses including only surgically treated patients^2,3,16,18^ reported favorable RTS outcomes after ACLR, particularly among competitive or pivoting athletes, but lacked nonoperative comparison groups.

Reported RTS rates after ACLR vary widely (16%-89%),^2,3,16,18,24,29,31,32^ while studies of nonoperative treatment have shown RTS rates between 46% and 68%.^4,27^ In our observational study, 77% of patients with ACLR and 72% of patients without returned to their preinjury sport. However, comparisons across studies are challenging due to differences in RTS definitions and heterogeneous study populations. For instance, some studies included only males^17^ or elite athletes,^19,24,30^ which may influence RTS rates. Our study included a heterogeneous population, with a significant difference between the 2 groups regarding preinjury Tegner level, in which a higher proportion of patients in the ACLR group were active in pivoting sports and thereby had a higher preinjury Tegner activity level. This may affect RTS rate in both directions, as lower preinjury activity may facilitate RTS, whereas highly active athletes may be more motivated to RTS. Our previous latent class analyses showed, however, that motivation was lowest in the subgroup of individuals participating in IKDC level 1 sports who were predominately male, with a mean age of 26 years, compared to both the subgroup of younger individuals participating in IKDC level 1 sports and the subgroup of older individuals who were predominately female and participating in noncontact sports.^4^ In addition, our risk analysis did not identify preinjury Tegner activity level as a significant factor when comparing Tegner scores 2 to 6 to Tegner scores 7 to 9 between the 2 groups. Within the predefined age categories, patients aged 26 to 40 years had a higher risk of not returning to sport in the ACLR group compared with the non-ACLR group. It should be noted that RTS outcomes may also be influenced by psychosocial, motivational, or social factors.^1^ For example, older patients may be physically capable of participating in cutting or pivoting sports but may choose not to return to their previous level due to lifestyle, work, or other social considerations. We attempted to collect information on reasons for not returning to sport, including psychosocial and lifestyle aspects, but only approximately 50% of patients responded at 2 years, which was deemed insufficient for formal analysis. Nonetheless, these factors may contribute to variation in RTS rate across age groups and should be considered when interpreting the results. Previous studies have reported younger age, male gender, and positive psychological response as predictors for higher RTS.^7^ Taken together, our results indicate that non-ACLR may be a viable option for selected patients and that ACLR is not universally required to achieve satisfactory RTS rates. Therefore, careful patient selection and shared decision-making might remain crucial to optimize functional recovery and RTS rates.

This study has several strengths. First, it is an observational, prospective cohort study, following patients with acute ACL injuries who were managed according to clinical routine practice. Because treatment decisions were not influenced by the study protocol, the results reflect actual outcomes and allow for the description of natural treatment pathways. To our knowledge, few previous studies have used this study design to compare the outcome of patients treated with and without ACLR. Patients were recruited from a variety of clinics, including both public and private clinics across different regions in Sweden, which enhances the generalizability and external validity of the findings. Additionally, new knee injuries and surgeries were systematically assessed, allowing for more accurate interpretations of functional outcomes. The study also includes extensive follow-up data for both treatment groups, with validated outcome measures such as the IKDC-SKF and Tegner activity scale.

The limitations of this study include the missing data at follow-up, which may introduce bias and affect the strength of the results. Age was analyzed using predefined categories. In the older non-ACLR group, nearly all patients returned to sport, resulting in minimal outcome variability and limiting the ability to detect meaningful differences between narrower age intervals. We did observe a higher dropout rate in the ACLR group, and within that group, fewer men responded. This means that missing data are not completely random and could potentially affect the results. However, the overall patterns in IKDC-SKF and RTS were consistent with the main findings, so we believe the conclusions are still informative, but should be interpreted with some caution. Moreover, as with all nonrandomized studies, there is a selection bias. In our study, the ACLR group had higher preinjury activity levels and younger age compared to the non-ACLR group. Therefore, between-group comparisons should be interpreted with caution. In this real-life study, we evaluated the clinical routine, which included shared decision-making for whether to undergo ACLR or not, and where patients were treated according to treatment recommendations.^7,12^ In addition, the study was not specifically powered to detect small between-group differences in RTS rates; therefore, the findings should be interpreted as comparative outcome estimates rather than proof of equivalence between treatments.

Another limitation is that a majority of patients undergoing ACLR received hamstring autografts; therefore, it remains unclear whether graft choice may have influenced the results. In Sweden, the most common graft choice is hamstring (82%).^5^ The study population was recruited within the Swedish health care system, and findings may not be directly generalizable to other health care settings or populations.

The findings of this study support a more individualized and nuanced approach to the management of ACL injuries. Although ACLR may facilitate return to preinjury levels of sport, particularly in athletes participating in pivoting or cutting sports, our results suggest that nonoperative treatment can also yield favorable functional outcomes in appropriately selected patients. In our risk analyses, preinjury Tegner activity level was not identified as a significant factor associated with the risk of not returning to sport within 24 months. This finding may, in part, be influenced by selection bias, as the ACLR group was younger and had higher preinjury activity levels compared with the non-reconstructed group. These results highlight the importance of considering patient-specific factors, such as activity demands, personal preferences, and patient characteristics, when determining the optimal treatment strategy.

## Conclusion

Both non-ACLR management and ACLR, determined through shared decision-making, resulted in comparable patient-reported knee function and RTS rates at 24 months, although the ACLR group was younger and had higher preinjury activity levels. Older age (26-40 years) was associated with a higher risk of not returning to sport among patients undergoing ACLR. Thus, selected patients with an ACL injury—particularly those who are older or have lower preinjury activity demands—may achieve satisfactory RTS without ACLR, although individual factors beyond age and activity level should also be considered. These findings support individualized treatment decisions based on age, activity level, and patient preferences.

## Supplemental Material

sj-docx-1-ajs-10.1177_03635465261451698 – Supplemental material for Patient-Reported Knee Function and Return-to-Sport Rates After Nonsurgical and Surgical Treatment of an Acute Anterior Cruciate Ligament Injury: Results From the NACOX Prospective Cohort Study Supplemental material, sj-docx-1-ajs-10.1177_03635465261451698 for Patient-Reported Knee Function and Return-to-Sport Rates After Nonsurgical and Surgical Treatment of an Acute Anterior Cruciate Ligament Injury: Results From the NACOX Prospective Cohort Study by Anna Croné, Håkan Gauffin, Henrik Hedevik, Anne Fältström and Joanna Kvist in The American Journal of Sports Medicine
