# Supplementary material for: Patient-Reported Knee Function and Return-to-Sport Rates After Nonsurgical and Surgical Treatment of an Acute Anterior Cruciate Ligament Injury: Results From the NACOX Prospective Cohort Study
Source: Am J Sports Med. 2026 Jun 8;54(9):2111–9. doi: 10.1177/03635465261451698 (PMC13354836; doi:10.1177/03635465261451698)
Supplement: sj-docx-1-ajs-10.1177_03635465261451698 – Supplemental material for Patient-Reported Knee Function and Return-to-Sport Rates After Nonsurgical and Surgical Treatment of an Acute Anterior Cruciate Ligament Injury: Results From the NACOX Prospective Cohort Study [file sj-docx-1-ajs-10.1177_03635465261451698.docx]

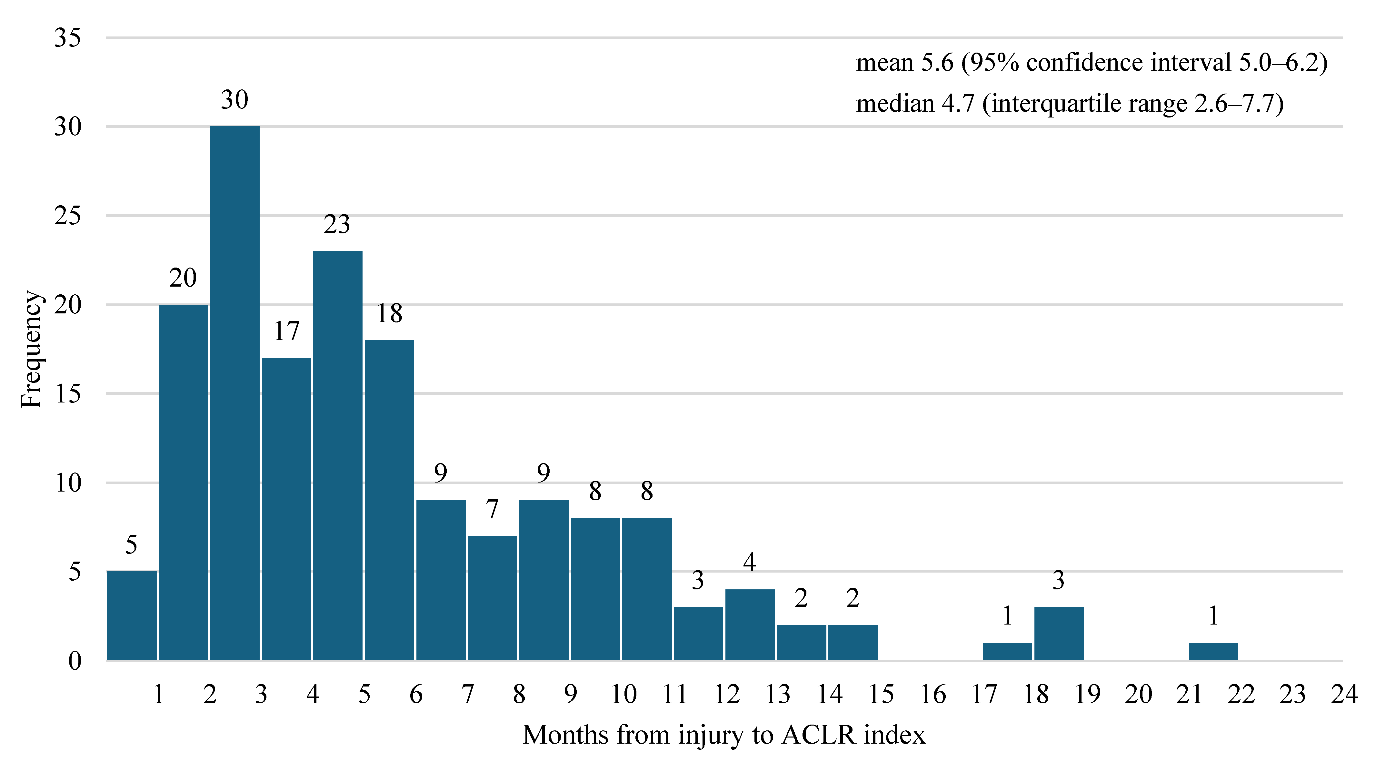


**Figure A1.** Diagram on frequency of the time of anterior cruciate ligament reconstruction (ACLR) up to 24 months.


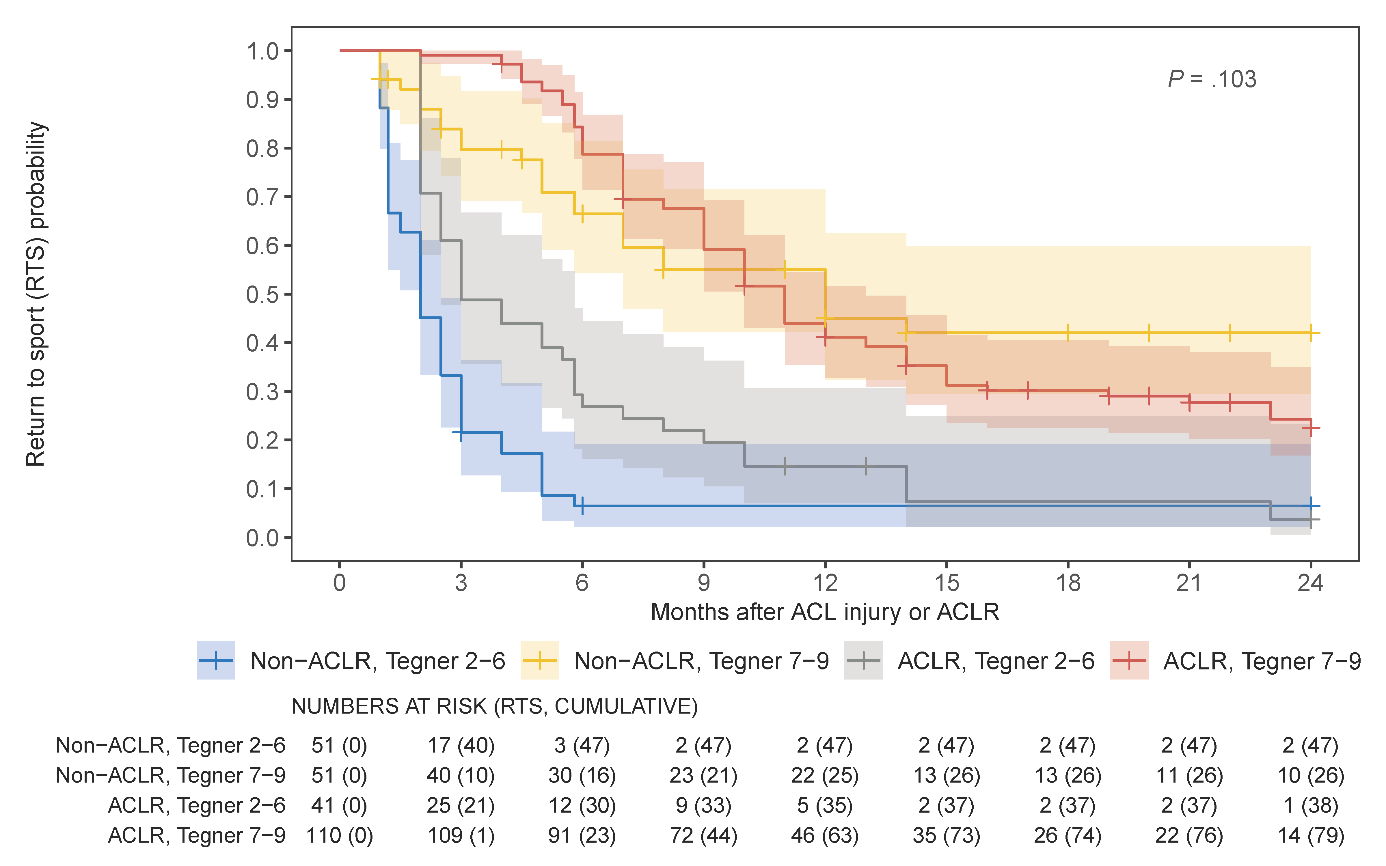


**Figure A2.** Kaplan-Meier curves on return to sport (same Tegner level or higher) within 24 months by group (non-ACLR and ACLR) and stratified by preinjury Tegner activity level


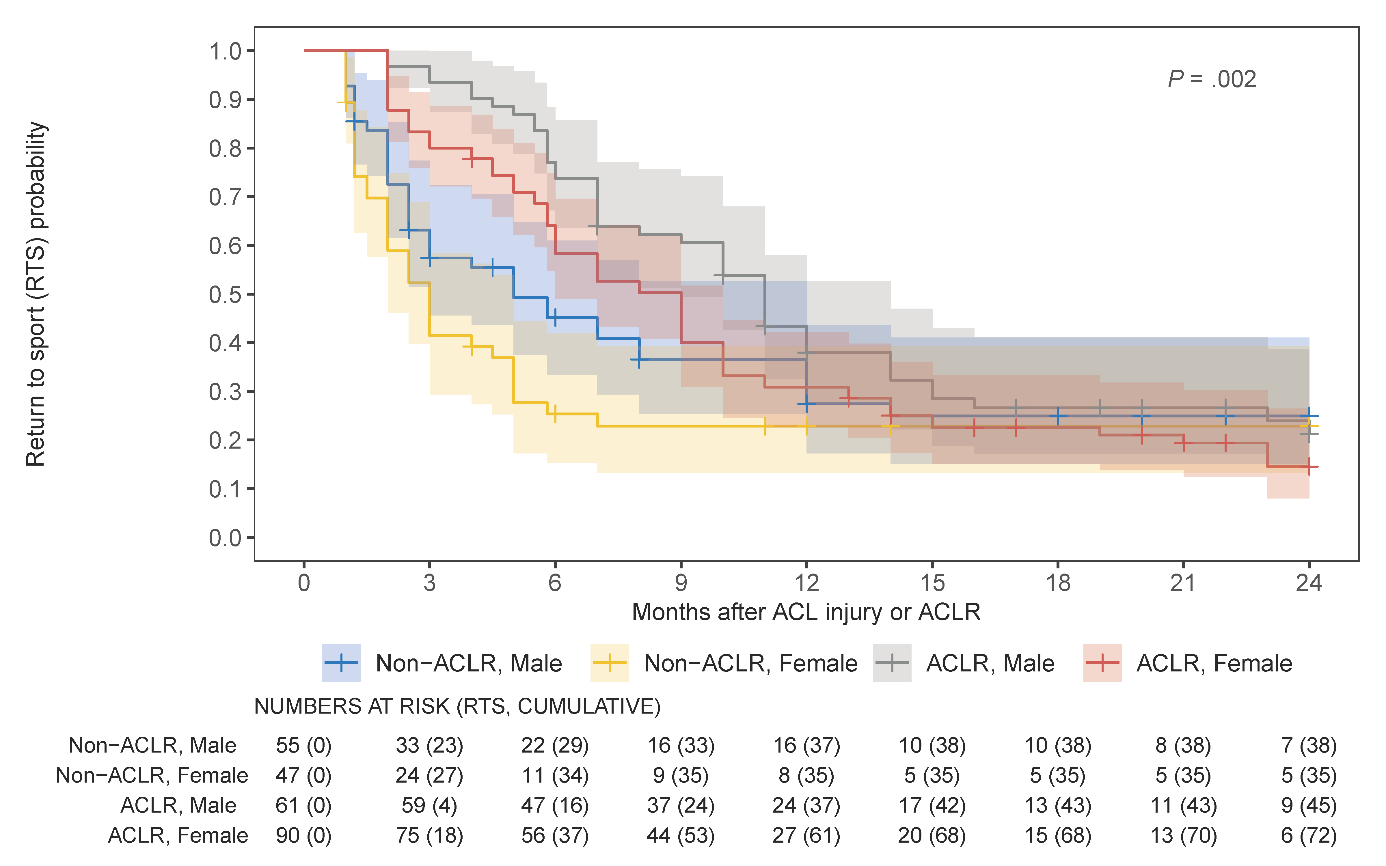


**Figure A3.** Kaplan-Meier curves on return to sport (same Tegner level or higher) within 24 months by group (non-ACLR and ACLR) and stratified by sex
